# Properties of Biocomposites from Rapeseed Meal, Fruit Pomace and Microcrystalline Cellulose Made by Press Pressing: Mechanical and Physicochemical Characteristics

**DOI:** 10.3390/ma14040890

**Published:** 2021-02-13

**Authors:** Tomasz Żelaziński

**Affiliations:** Department of Production Engineering, Institute of Mechanical Engineering, Warsaw University of Life Sciences—SGGW, Nowoursynowska 164, 02-787 Warsaw, Poland; tomasz_zelazinski@sggw.edu.pl

**Keywords:** biocomposites, process parameters, fruit pomace, mechanical engineering, hydraulic press, flexural strength, thermal analysis

## Abstract

This paper presents the results of research on biocomposites made of the mixture of post-extraction rapeseed meal, microcrystalline cellulose and various fruit pomace (chokeberry, blackcurrant, apple and raspberry pomace). The biocomposites were made in the process of mechanical thickening by means of a heated mould (die and stamp) which is located between two heating elements installed on a hydraulic press. The presented research combines mechanical engineering and material engineering issues. The physical and mechanical tests of obtained biocomposites included mechanical strength measurements, thermogravimetric analyses (TGA), colour change tests and scanning electron microscopic (SEM) tests of the internal structure after breaking the sample. In addition, Fourier transform infrared spectroscopy (FTIR) tests were carried out. Generally, the bend tests and Young’s modulus were significantly increased, for example, biocomposites with an addition of chokeberry pomace had the flexural strength higher by approximately 25% in relation to the primary sample. Furthermore, it is interesting to note the increase of water contact angle of these biocomposites by 40% in relation to the primary sample. The research indicates the potential for using fruit pomace for the needs of biocomposite production.

## 1. Introduction

Environmentally oriented activities observed in the world economy will force the search for new solutions, limiting the use of plastics in the agro-food industry [1]. At the same time, in line with growing awareness of people of the adverse effects on the environment from the technology using fossil fuels, each activity aimed at improving the surrounding environment is well-perceived by the society [2,3,4,5]. Present trends and increasing demand mean that biodegradable products do not only include foils, disposable plates, drinking straws or other small objects for everyday use. Researchers increasingly strive to obtain biocomposites of a constructional nature that could be used in various industrial sectors, e.g., automotive industry (vehicle interior elements), construction industry (planks, beams), furniture industry (boards) and others [6,7]. At present, these trends are widely promoted all around the world, which opens the possibilities of implementing such innovative products on a global level. The use of raw materials, which are completely of natural origin and consistent with sustainable development and “zero waste” rules, is an important criterion in the production of modern biocomposites [8,9]. This type of raw materials includes various by-products of earlier technological processes related to agricultural and food processing. They can include, e.g., fruit and vegetable pomace from juice production, spent grains from beer production, oil cake after oil extraction and many others. These raw materials are usually rich in a number of natural binding components (lignocelluloses, polysaccharides, proteins, etc.). After suitable treatment, they may continue to be a valuable material for the manufacture of new biodegradable products intended for a range of new applications [10]. The rational use of vegetable waste materials for the needs of the biocomposite manufacture is therefore fully reasoned and prospective [11]. The essence of the modern biocomposite production is to find proper components from which a finished product can be directly made. It is also essential to match appropriate parameters of mechanical pressing or compacting processes, as is indicated by Lisowski et al. [12]. Despite a number of advantages of biodegradable materials, in many cases, the quality of these products is not satisfied. Generally, some researchers say that unsatisfied physical and mechanical properties are one of the main limitations regarding the manufacture of biodegradable materials [13]. Therefore, the solution seems to be the search for new components which can improve the properties of such biocomposites.

Raw materials, the potential of which is not sufficiently used, can include materials rich in proteins. These involve pomace after production of vegetable oil, from which so-called post-extraction meal is manufactured. The meal contains 30–38% of vegetable protein and approximately 20% of crude fibre on average. As it results from the published references, the attempts to manufacture biocomposites based on high-protein raw materials generally came out well. This was confirmed by the tests carried out on soybean materials and grape marc [14]. The author stated that an addition of proteins had a particularly favourable effect on the improvement of mechanical properties of biocomposites. The authors of Reference [15] stated similarly, however, that they connect the improvement of the mechanical properties with crosslinking properties of proteins. According to Prochoń and Ntumba [16], sulphur protein amino acids containing rich rapeseed protein have particularly good crosslinking properties. Such properties of proteins show the wider possibilities of using them, e.g., as binders or adhesives, which significantly expands the possibilities of high-protein raw material applications [17]. The proteins also demonstrate antioxidant activities, which is associated with combining active forms of oxygen and creating reactions of polymer chains, which is undoubtedly their positive feature [18]. Furthermore, heat-treated proteins are characterised by increased thermal resistance due to a large number of atoms in the polypeptide molecule which can provide tangible benefits to the heat-treated products [19]. In addition to many favourable properties of proteins, it should be noted that the proteins are generally available and more ecological than synthetic binders. According to the other authors’ research, it is possible to use protein raw materials to manufacture thickened stable plastics, however, such products can be fragile [20]. In line with the other references, the properties of such products can be improved by adding cellulose raw materials, such as microcrystalline cellulose (MCC) [21]. Such a material is fully biodegradable and can perfectly complement the modern biocomposites [22].

For ecological reasons, it is also justified to combine protein raw materials with other significantly cheaper biodegradable raw materials. This may enrich the composition of such materials and improve physical and mechanical parameters of obtained products at the same time [23]. Such products, which can include fruit residues after juice extraction processes, are waste materials, and are difficult to manage. It is generally accepted that fruit pomace is particularly rich in vegetable fibres, the content of which can exceed even 70% in the dry mass. However, it generally depends on the pomace type which is affected by the amounts of certain components: husks, stones/kernels and woody parts. Taking account of the fact that the vegetable fibre composition includes mainly cellulose, hemicellulose, lignin and pectin, fruit pomace can be a raw material for biocomposites. Those products, after thickening, will be a homogenous durable material, which is pointed out by the studies of other researchers [24]. Chokeberry, currant, apple and raspberry pomace can be included to the promising raw materials, which due to their properties, could enrich the composition of modern biocomposites. According to Górecka et al. [25], raspberry pomace includes 24.2% of cellulose, 6.00% of hemicellulose and 24.60% of lignin. According to Reference [26], the dry mass of chokeberry pomace contains: 33.14% of cellulose, 32.8% of hemicellulose, 23.03% of lignin, 7.52% of pectin and 4.23% of other components. The dry mass of blackcurrant pomace includes 7.92% of cellulose, 14.69% of hemicellulose, 73.8% of lignin, 2.48% of pectin and 1.11% of other components. The dry mass of apple pomace consists of 43.17% of cellulose, 24.27% of hemicellulose, 11.76% of pectin, 20.34% of lignin and 0.46% of other components.

Taking the above into account, the combination of high-protein raw material (rapeseed meal) with pomace (with a different fibre composition) will make it possible to explore new possible applications for these components. An added value of research is also the manufacture of a new type of biocomposites based mainly on agricultural and food industry by-products.

The purpose of this paper is therefore to verify the possibilities of using post-extraction rapeseed meal with a MCC addition and various fruit pomace to produce biodegradable composite materials by the hot-pressing method.

## 2. Materials and Methods

### 2.1. Materials

The material used to produce biocomposites included post-extraction rapeseed meal, dried fruit pomace (chokeberry, currant, apple and raspberry pomace) and microcrystalline cellulose (MCC). The post-extraction rapeseed meal was purchased from a local market (Mar-Rol, Jarocin, Poland). The basic chemical composition (% in dry weight) was as follows: 39.6% of total protein, 13.7% of crude fibre, 7.8% of crude ash, 2.5% of crude fat and 36.4% of other ingredients (manufacturer data). Fruit pomace was delivered by a fruit processing company (Greenherb, Łańcut, Poland). The company also dried the pomace by using a drum dryer with heat exchanger at a temperature of 100 °C. Dried fruits comprised pomace obtained after juice extraction from fruits (country of origin—Poland: chokeberry (melanocarpa (Michx.) Elliott) black currant (Ribes nigrum ‘Tiben’), raspberry (Rabus idaeus ‘Polana’) and apple (Malus domestica ‘Chmpion’, Malus domestica ‘Lobo’, Malus domestica ‘Ligol’). An addition of microcrystalline cellulose (MCC) (Cellulose Powder, Cotton linters), 20 µm type (Sigma Aldrich, Saint Louis, MO, USA), was used in the composition of the mixture for manufacture of biocomposites. The percentage of components intended for manufacture of biocomposites along with process temperature and sample acronym are specified in Table 1. The basic composition of each mixture was the sum of three components (%wt—weight percentage), e.g., fruit pomace—10 wt% + rapeseed meal 83 wt% + microcrystalline cellulose (MCC) 7 wt%, etc. The control sample consisted only of rapeseed meal and MCC. Each sample was produced at the process temperatures of 130 and 160 °C. In this way, 26 samples of biocomposites were produced for further research. The paper also uses markings which facilitate description of results, e.g., (10_130), (20_130), (30_130), (10_160), (20_160), (30_160). The first part of the abbreviation means the percentage of specific fruit pomace in the mixture (10, 20, and 30 wt%), and the second part means the process temperature (130 or 160 °C).

### 2.2. Biocomposite Production

The biocomposites were made of post-extraction rapeseed meal mixtures with an addition of various fruit pomace (chokeberry, currant, raspberry and apple pomace). The 7 wt% microcrystalline cellulose (MCC) addition was used as a strengthening component in each mixture. The rapeseed meal and pomace were shredded by a MKM 6000 impact mill (BOSCH, Gerlinge, Germany). The material was shredded during 25–30 s until the particles below 1.6 mm were obtained (during milling, the material was agitated twice for approximately 2 s). The granulometric composition of individual mixture components were checked by a LPzE-2e sieve screen unit (MULTISERW-Morek, Brzeźnica, Poland). The sieves with a size of 1.6, 1.0, 0.8, 0.5, 0.25, 0.1, <0.05 mm compliant with DIN ISO 3310-1:2017-11 were used in the screen unit column [27]. The screening time was 10 min, vibration amplitude was 60 and frequency was 2 Hz. A detailed distribution of raw material particles after shredding was presented in Table 2. The material for the manufacture of biocomposites was prepared in a plastic container with a capacity of 400 mL and mixed by a CAT 30 mechanical stirrer (CAT, Deerfield, IL, USA) at a speed of 250 rpm for 60 s. The material moisture content, after mixture preparation, was 9.2% (±0.2%).

**Figure 1:**
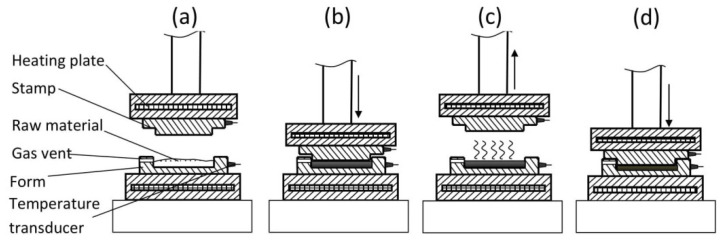
Biocomposite production process: (**a**) material load, (**b**) initial pressing, 5 MPa during 30 s, (**c**) evaporation of water, 10 s, (**d**) main pressing, 20 MPa, time 5.5 min.

The samples were made on a test bench consisting of a hydraulic press with a pressing force of maximum (max.) 150 KN (FR–5014, producer, Farys, Poland). Upper and lower heating plates with a thickness of 30 mm, with internal heating coils with a total power of 1600 W, were installed on a basis and piston rod. The temperature of heating plates was adjusted separately for the upper place and lower plate by means of heater controllers with an accuracy of ±0.1 °C. The heating plates were used to heat a metal mould used in the biocomposite pressing process (Figure 1). The mould made it possible to manufacture square biocomposite plates with dimensions of (length 90 mm, width 90 mm and a thickness of 5 mm). Each time, in order to produce a single sample, the mould was poured with 80 g of prepared material. The samples were manufactured at two thickening process temperatures, i.e., at 130 and 160 °C. The thickening process was carried out in two stages. In the first stage, the raw material was subjected to 5 MPa stress for approximately 30 s. Then, the die stamp was lifted for approximately 10 s (at this time, water rapidly evaporated). In the second stage, the material was pressed once again, but with the increased pressure equal to 20 MPa for 5.5 min. The material obtained in such a way was kept at room temperature for 5 h.

The process parameters (temperature, exposure time) were selected on the basis of preliminary studies and literature data. The selected temperature range of the process resulted from the significant amount of protein in the raw material. To obtain the stickiness of the protein, it is necessary to denature it. The above pressing method was chosen because it is one of the simplest methods used in the production of such materials. It may be relevant for the further commercial application of such products (e.g., production of consumables).

### 2.3. Mechanical Properties

The material strength was determined by the three-point flexural method and the Young’s modulus (YM) was defined. The flexural strength (FS) parameters of biocomposites were determined in accordance with PN-EN ISO 178:2011 [28]. The distance between supporting beams was 50 mm. The radius of the loading element was 5 mm. The samples were cut out by mechanical treatment. An INSTRON 8802 universal strength testing machine was used for strength tests (Instron, Norwood, MA, USA).

### 2.4. Water Contact Angle

The water contact angle values were determined by a so-called sitting drop method. The outline of a drop applied on the biocomposite surface was analysed and the internal tangent inclination angle to the horizontal surface at the point of contact of drop and material was determined in accordance with Giri et al. during the measurements [29]. The tests were carried out on the measurement bench consisting of adjustable table, syringe for dosing drops of distilled water and A2500-14uc, 5 megapixels (Mpix) camera (producer: Basler, Ahrensburg, Germany). The drop volume was 15 µL, and water temperature was 23 °C. Pictures were taken for up to 1 s after drop application by Pylon Viewer software (producer: Basler, Ahrensburg, Germany). The pictures, taken in such a manner, were introduced as a raster image to Autodesk Autocad Mechanical 2019 software, product version: 23.0.46.0, where the water contact angle values were determined.

### 2.5. Colour Analysis

A change of the colour was analysed on the basis of pictures taken by an Optatech STX stereomicroscope equipped with a 5 megapixels (Mpix) colour camera and light emitting diodes illuminator (LED) with a colour temperature of 7000 K (Opta-Tech, Warsaw, Poland). The camera was calibrated by performing a white balance by means of Minolta S no. 1863310 white chart. The research used the L*, a*, b* colour space (L*—brightness, a*—colour from green to magenta, b*—colour from blue to yellow). The values of individual components of the colour were read by a histogram function using CorelDRAW Home and Student X7 Version 17.1.0.572 (Corel Corporation, Ottawa, Canada). The colour changes of biocomposites Δ*E* due to the process temperature difference were calculated in accordance with the following Equation (1):

(1) $$\Delta E={\left[{\left(\Delta L^{*}\right)}^{2}+{\left(\Delta a^{*}\right)}^{2}{\left(\Delta b^{*}\right)}^{2}\right]}^{1/2}$$

where ΔL*, Δa*, Δb* represent the changes in the colour value Δ*E* after increasing the process temperature from 130 to 160 °C.

### 2.6. Microscopic Analysis

The structure of obtained materials was analysed using the SEM HITACHI S-3400N scanning electron microscope (SEM, Tokyo, Japan) with an accelerating voltage of 20 kV in low vacuum conditions under 70 Pa. The composite fracture and external surface were analysed.

### 2.7. Thermogravimetry Analysis (TGA) and Derivative Differential Thermal Analysis (DTA)

The thermogravimetry analysis (TGA) and derivative differential thermal analysis (DTA) were carried out with the use of Q50 TGA V20. 13. Build 39 (TA Instruments, New Castle, DE, USA). The sample with a mass of 50 mg was heated at a speed of 10 °C min^−1^ from room temperature to 700 °C. The nitrogen flow was 40 mL min^−1^.

### 2.8. Fourier Transform Infrared Spectroscopy (FTIR)

The infrared spectrum (FTIR) of biocomposites was analysed by means of a spectrometer-model Spectrum 2000 (Perkin-Elmer, Waltham, MA, USA). The spectra were recorded at a resolution of 4 cm^−1^ within the range of 400–4000 cm^−1^. The measurements were conducted at room temperature. In order to improve the accuracy of the research, 32 scans were made for a single sample.

### 2.9. Statistical Analysis

The findings obtained (strength tests, water contact angle, colour changes) were subjected to statistical treatment in the STATISTICA 2013 version 13.3 software (TIBCO Software Inc., Palo Alto, CA, USA). The results were presented as average values (*n* = 5) ± standard deviation (SD). The Shapiro–Wilk test was used to test the normality of the data. The differences were considered relevant at the confidence level of 95% (*p* < 0.05) in one-way analysis of variance (ANOVA) examination with a post-hoc Tukey’s test. Statistically significant and not significant differences are presented by means of capital and small letters placed above the bars of the graphs. Various case letters (e.g., a, b, c, d…) located at the results indicate relevant differences between the percentage of pomace share in the sample. Various capital letters (e.g., A, B, C, D…) indicate relevant differences between the individual sample biocomposites with the same percentage share of components. The same letters, for example, (a, a) or (B, B) means no statistical significance (between homogeneous groups.

## 3. Results

### 3.1. Flexural Strength and Young’s Modulus

**Figure 2:**
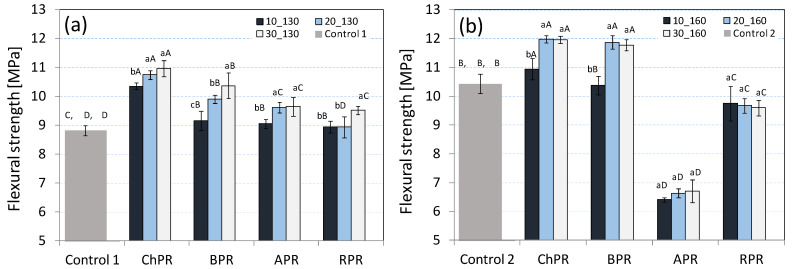
Average flexural strength values, for all biocomposites with each share of used pomace: (**a**) sample produced at temperature of 130 °C, (**b**) sample produced at temperature of 160 °C.

**Figure 3:**
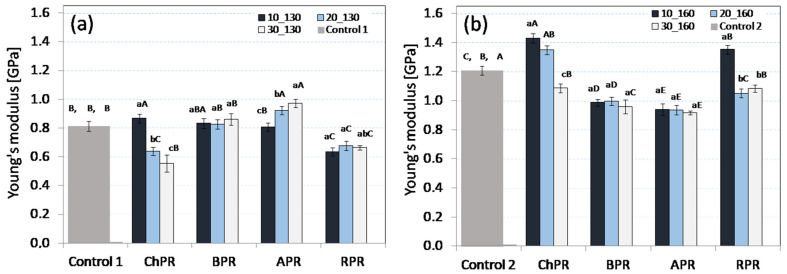
Average Young’s modulus, for all biocomposites with each share of used pomace: (**a**) sample produced at temperature of 130 °C, (**b**) sample produced at temperature of 160 °C.

On the basis of the obtained results, it was found that the flexural strength (FS) of the produced biocomposites was different depending on the type of pomace and its percentage share in the mixture (Figure 2a,b). It was stated that the materials with an addition of chokeberry pomace (ChPR) were characterised by the largest FS strength, which was observed at the production process temperatures of 130 and 160 °C. Comparing the results to the rapeseed and microcrystalline cellulose (MCC) sample (Control 1), the use of process temperature of 130 °C allowed the improvement of the strength parameters of biocomposites. At this process temperature, the strength increased each time in tandem with the share of fruit pomace in the mixture. The use of the process temperature of 160 °C allowed the improvement of the strength parameters of ChPR and BPR biocomposites (chokeberry and currant pomace share at the level of 20 and 30 wt%), where the maximum stresses during binding were from 11.8 to 12.1 MPa. The same temperature in case of APR and RPR biocomposites caused the relevant reduction of strength parameters in relation to the control sample (Control 2). In this case, the low-strength values could be associated with too rapid water evaporation when the mould stamp is lifted, as a result of which small pores (internal and external) formed. A similar phenomenon is observed, inter alia, when the raw material passes from the area of high pressure to the area of atmospheric pressure in the extrusion process of vegetable raw materials [30]. Generally, the observed increase of the strength parameters of composites in tandem with the increase of pomace share can be associated with a fibrous structure of the raw material and cross-linking properties protein the rapeseed meal. This is confirmed by research [16,17]. So, the strength increase can be associated with the increase of the quantity of fibre in the mixture which was delivered together with fruit pomace. The strengthening properties of vegetable fibre used in biocomposites were pointed out by, inter alia, such researchers as those of References [31,32,33]. The strength increase can be also affected by the Maillard reactions, which support the crosslinking process of proteins included in the rapeseed meal [34]. This can explain the strength increase of obtained ChPR and BPR materials in line with the increase of temperature. However, the comparable strength parameters of these two biocomposites are surprising because as it was presented in Reference [26], these products differ significantly, especially in the lignin share. This lignocellulose component is now a very interesting raw material from which nanoparticles are also obtained, useful in the production of advanced materials [35]. Such observations may open new opportunities with regard to research associated with the use of lignin as components of new biocomposites. The results of Young’s modulus presented in the graphics (Figure 3a,b) show that the elasticity increase compared to the control sample (Control 1, Control 2) was observed only in case of the ChPR biocomposite (chokeberry pomace 10 wt%, temperature of 130 °C), APR (apple pomace 20 and 30 wt%, temperature of 130 °C), and in case of ChPR samples (10 and 20 wt%, temperature of 160 °C) and RPR (10 wt%, temperature of 160 °C). In other cases, the elasticity of samples was less or not statistically significant. In this case, a higher elasticity coefficient is advantageous because the obtained values are close to other biodegradable products.

For comparison, the flexural strength of apple pomace and poly (butylene succinate) plates is about 45 MPa, and Young’s modulus is about 2 GPa [24]. Another example is biocomposites made of polylactic acid (PLA), the flexural strength of which is about 150 MPa, and the Young’s modulus is about 3 GPa [36]. PLA is today a typical commercial biodegradable material for various applications. The samples produced during the research have quite good elasticity. For some applications, however, they may require increasing the strength parameters.

### 3.2. Water Contact Angle

**Figure 4:**
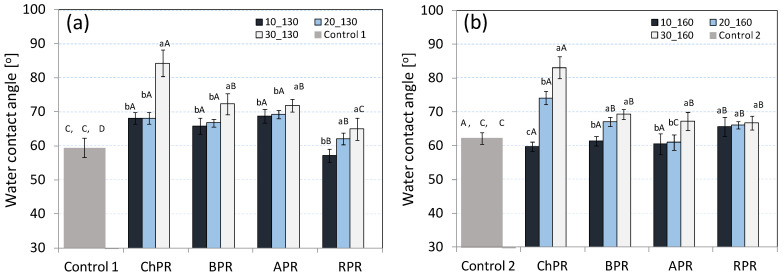
Average water contact angle, for all biocomposites with each share of used pomace: (**a**) sample produced at temperature of 130 °C, (**b**) sample produced at temperature of 160 °C.

The tests of water contact angle (Figure 4a,b) were carried out due to the fact that the wettability is the value which defines the use of biocomposites as biodegradable products for everyday use (e.g., plates, cups, saucer, cutlery, etc.). It was found that all the obtained biocomposites reached the water contact angle at the level of 59–84°, which indicates their hydrophilic nature [37]. However, it was stated that the increase of the pomace share in the samples up to 30 wt%, regardless of the used biocomposite production temperature, caused the increase of water contact angle. This means that the hydrophobicity of these products increased. This increase can be associated with adding significant quantities of lignin, recognised as hydrophobic, being in the composition of every pomace [38]. The highest water contact angle was stated for ChPR (84.2°) and BPR (73.1°) samples with an addition of 30 wt% of pomace. According to the literature data, a hemicellulose, generally recognised as hydrophilic, can be a factor limiting the hydrophobicity increase of materials [39]. At the same time, this impact can be limited through combining the remains of fats from used raw materials [40]. Such thesis can explain the least wettability of ChPR and BPR samples. The increase of the process temperature up to 160 °C caused a slight decrease of water contact angle of materials with the 30 wt% share of ChPR, APR and BPR pomace. This can be explained by the beginning of chemical transformation causing an initial decomposition of lignocellulosic components [41]. This may be related to the softening of the pectin matrix, which is a binder for cellulose fibres, as indicated [42]. The results of water contact angle are similar to the exemplary biocomposites reinforced with nanocellulose and microcrystalline cellulose (MCC) [29].

For comparison, the most hydrophobic biodegradable materials have a contact angle as high as 158° [43]. Pure PLA, on the other hand, has a contact angle of only 75° [44]. Taking into account the above, the tested biocomposites may require improvement of this parameter.

### 3.3. Colour Analysis

**Figure 5:**
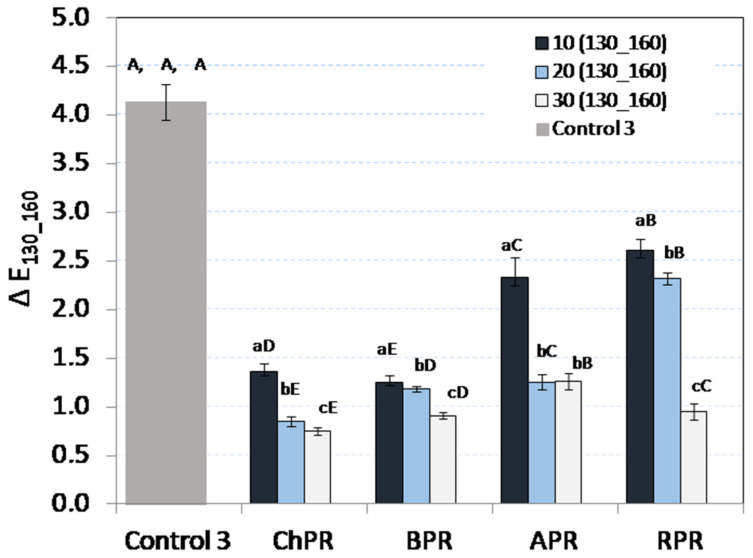
Average colour change values, Δ*E*, resulting from increase in process temperature from 130 to 160 °C.

It was found that the obtained biocomposites were dark, in shades close to brown and black (Table 3). This is due to the low values of product luminosity, L* (9.04–12.09 wt%), and the values of coefficients a* and b*, which range between −0.18 and 2.49 on the Lab colour space scale. It was also stated that the luminosity of materials manufactured at a temperature of 130 °C decreased with the increase in the pomace share. A similar tendency was observed in case of materials manufactured at a temperature of 160 °C, however, this concerned only ChPR, APR and BPR biocomposites. While analysing the parameters of colour a* and b* for APR and BPR biocomposites, it was found that the values tended to zero on the Lab colour space scale along with the share increase of chokeberry and currant pomace. So, this explains the share increase of black shades in these products. In the case of ChPR and BPR biocomposites, the dark colour may also be caused by the content of anthocyanins present in chokeberry and black currants [45]. This pigment also contributes to the more intensive colouring of bright particles of mixture components. It was stated as well that the colour changes are affected by the temperature increase in the sample thickening process. In this case, the darker colour may result from protein denaturation and the Maillard reaction (browning) in the surroundings where the process temperature is increased up to 160 °C [46]. Figure 5 presents the Δ*E* differences between the colour of materials produced at a temperature of 130 and 160 °C. Research indicated that the most intensive changes, at the level of 1.25–2.62, appeared in the samples with an addition of 10 wt% of pomace. The share increase in fruit pomace up to 30 wt% caused the decrease in the value of this indicator in each case. It was found that in case of ChPR and APR biocomposites, the changes in colour were least intensive, which indicates the increased resistance of these materials to the colour changes while the sample is heated. At the same time, it was also stated that the sample based on rapeseed meal and MCC (Control 3) is least resistant to the process temperature increase. In this case, the colour difference is 4.2 and it is clearly perceptible even with the naked eye.

### 3.4. Microscopic Analysis

**Figure 6:**
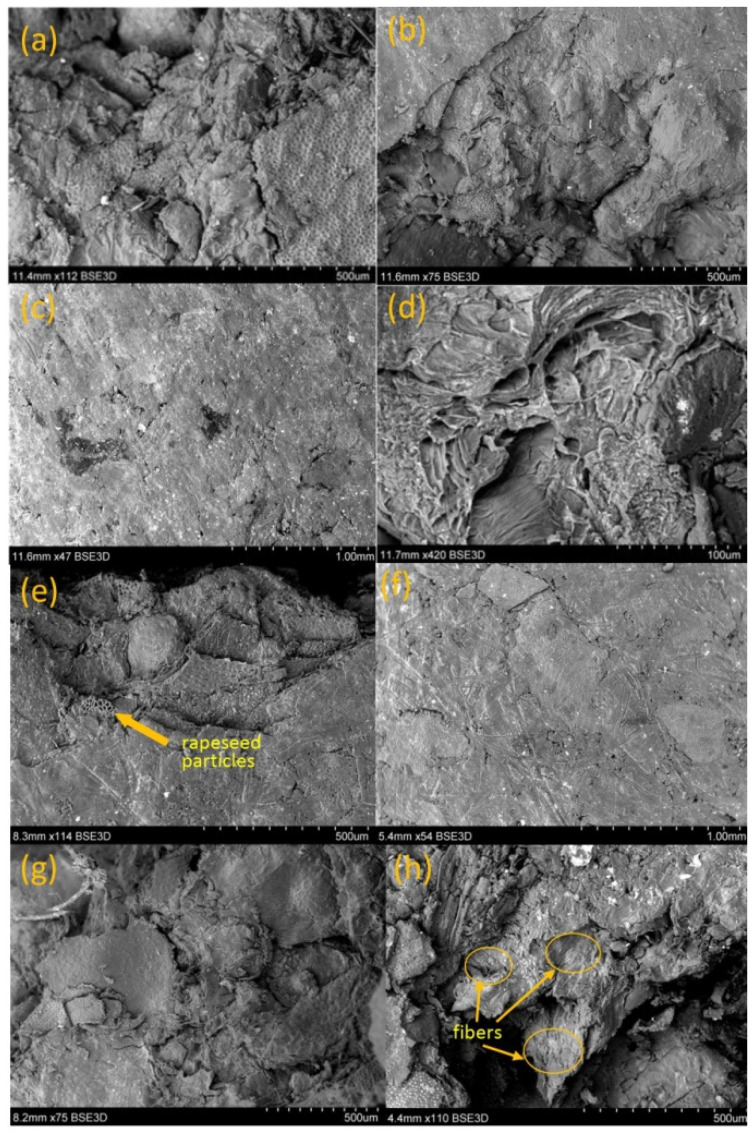
Scanning electron microscope (SEM) images of selected biocomposite samples: (**a**) sample fracture site ChPR (30_130), (**b**) sample fracture site ChPR (30_160), (**c**) sample surface ChPR (30_160), (**d**) close-up of the fibre embedded in the matrix ChPR (30_160), (**e**) sample (control 1), (**f**) APR sample area (30_160), (**g**) sample fracture site RPR (30_130), (**h**) sample fracture site APR (30_130).

The SEM microscope analysis was carried out in order to assess the impact of used additions on the changes in the internal and external structure of obtained biocomposites. The utilitarian purpose was to recognise the changes in the structural features of biocomposites after the sample fracture, which could have a direct impact on the strength parameters of the samples. It was found that a characteristic feature of all the samples was a structure consisting of raw material particles flattened to a varying degree (Figure 6a,b,e,g). Such a structure was most evident in case of the sample (Control 1) made of rapeseed and microcrystalline cellulose MCC (Figure 6e), as well as in case of other samples manufactured at a temperature of 130 °C. The process temperature increase up to 160 °C contributed to the better fluidisation of material structure (Figure 6b,d), which also could be affected by better denaturation of protein included in the raw material. Furthermore, all the obtained samples were characterised by a smooth external surface with visible small micropores (Figure 6c,f). The pores are usually formed due to rapid water evaporation and they are the result of rapid changes in pressure. The limitation of porosity is usually a challenge when planning the production process of biocomposites. However, it can be a positive feature when a protective layer is applied on such a type of products. While analysing the points of sample fractures, it can be observed that the introduction of an additional vegetable fibre (in the form of fruit pomace) makes numerous material extensions/elongations at the point of sample fracture (Figure 6h). This can be observed especially in case of ChPR and BPR (pomace 30 wt%, temperature 130 °C). According to Picard [24], such a situation occurs when the material consistency is disturbed, which makes it easier to pull out fibres under the effect of a mechanical force. In turn, the consistency disturbances can be associated with hydrophobic and hydrophilic reactions (sorted fibre), which is indicated by other authors [47]. However, such a phenomenon, in the performed strength tests, improved the strength parameters of samples, in particular, ChPR and BPR samples. Therefore, the pomace addition improved the quality of phase-to-phase combinations between the used raw materials. The increased process temperature caused better consolidation of obtained materials (Figure 6b,c) and further improvement of phase-to-phase interactions of consolidated materials at the same time. Such a material structure could cause better stress dissipation during sample fracture and affect the improvement of mechanical properties of biocomposites, which was also stated in Reference [48]. To understand better the interactions between the fibres and the matrix, the distribution of raw material particles is presented (Table 2). Based on this analysis, it can be seen that the share of 0.5–1.6 mm particles accounted for more than 40 wt%. Less than 9% were smaller than 0.25 in size.

### 3.5. Thermogravimetry Analysis (TGA) and Derivative Differential Thermal Analysis (DTA)

**Figure 7:**
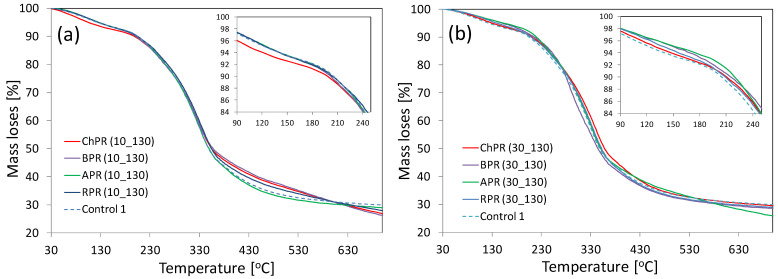

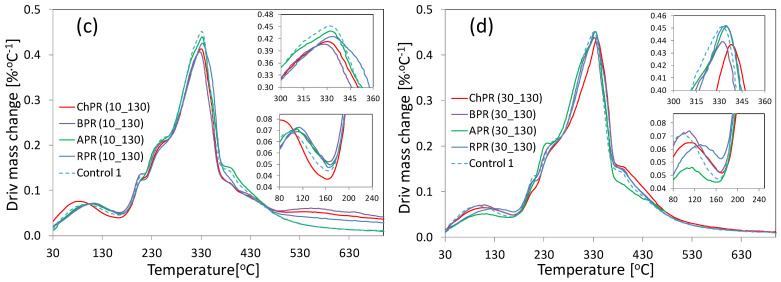
Thermogravimetric analyses of biocomposites: (**a**) mass loss of biocomposites containing 10 wt% of pomace, (**b**) mass loss of biocomposites containing 30 wt% of pomace, (**c**) derivative mass change of biocomposites containing 10 wt% of pomace, (**d**) derivative mass change of biocomposites containing 30 wt% of pomace.

Graphics (Figure 7a–d) present the results of thermogravimetry analysis (TGA) and derivative differential thermal analysis (DTA). The TGA was carried out for the share of pomace equal to 10 and 30 wt%, manufactured at the process temperature of 130 °C. It was found that in both cases, the mass loss took place in five main stages. The first temperature stage (30–170 °C) corresponds mainly to water evaporation from the sample. The mass losses in this stage ranged between 6.17% (BPR sample) and 7.63% (APR sample), for biocomposites with the 10 wt% share of pomace. The significant reduction of evaporated water was observed in case of BPR, APR and RPR composites with an addition of 30 wt% of pomace. The ChPR biocomposites, regardless of the amount of chokeberry pomace addition, were characterised by the mass loss at the level of 7.42–7.62%. The mass loss in the second zone ranged between 4.28% (ChPR with 30 wt% of pomace) and 5.42% (RPR with 10 wt% of pomace) and was associated with softening, mainly of cellulosic components, which is also indicated by Kamdem et al. [41]. The further mass loss in the temperature range of 220–260 °C could be due to degradation of hemicellulose and the beginning of degradation of fats contained in post-extraction meal. The next stage of temperature range (260–400 °C) covers mainly cellulose degradation [49]. The BPR sample (30 wt% of pomace) was significantly characterised by the highest thermal resistance in this process stage. This can be justified by the increased thermal resistance of lignin in relation to cellulose and hemicellulose contained in the raw material, which is indicated by Lisowski et al. and Kim et al. [12,50]. In the last temperature zone (400–600 °C), the mass loss was caused by depolymerisation and degradation of biocomposite components. In this stage, the mass loss is associated with the acetylated degradation of components of high molecular mass and other processes [51]. It was also found that the highest value of temperature, i.e., 152.7 °C, at which a 5% mass loss was recorded, was identified in the APR biocomposites (30 wt% of pomace), and the lowest value of temperature, i.e., 104 °C, was identified in the ChPR materials (10 wt% of pomace). A 50% mass loss was recorded in the temperature range between 344 and 358 °C, which also corresponded to the highest values of the mass derivative (Figure 7c, d). This is due to the high content of cellulosic components in the raw material composition of biocomposites.

### 3.6. Fourier Transform Infrared Spectroscopy (FTIR)

**Figure 8:**
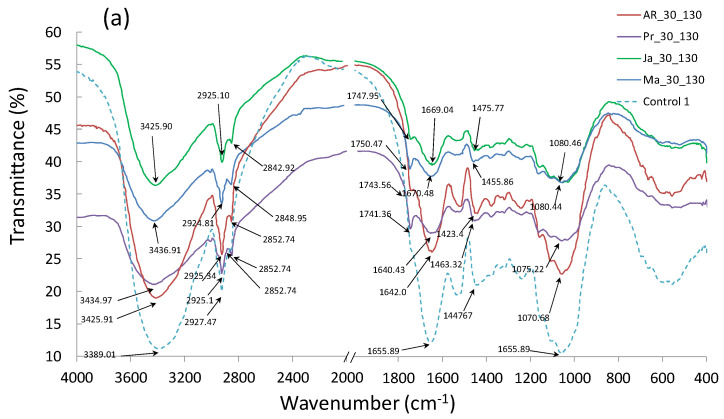

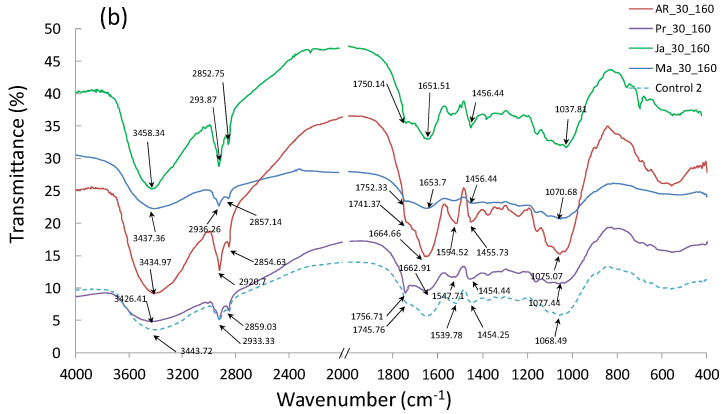
Fourier Transform Infrared Spectroscopy (FTIR): (**a**) tests of biocomposites containing 30 wt% of pomace, produced at process temperature of 130 °C, (**b**) tests of biocomposites containing 30 wt% of pomace, produced at process temperature of 160 °C.

The FTIR analysis was conducted for biocomposite samples manufactured at temperatures of 130 and 160 °C, with the highest pomace share of 30 wt% (Figure 8a,b). It was found that the obtained spectra showed a similar distribution, and the observed vibration types were typical for cellulose-rich organic materials, which was confirmed by References [24,52]. The use of additions of various fruit pomace did not contribute to formation of new groups of compounds—only a small shift in the spectrum transmittance distribution was observed. However, comparing the spectra obtained on the basis of the tests of samples manufactured at temperatures of 130 and 160 °C, a slight transmittance increase for hydroxyl bonds (-OH) was stated, in tandem with the process temperature increase. It was also found that the obtained results can be divided into three main areas. The first of them is between 2800 and 3500 cm^−1^, where the observed peaks can be associated with tension vibrations of bonds of methyl groups (O-H). The vibrations in the second area (1500–2200 cm^−1^) indicate the presence of groups of compounds containing double bonds (C=C, C=O). The vibrations typical of the fingerprint range were observed in the third region, where the bands correspond to deformation vibrations and originate mainly from single C-O bonds.

## 4. Conclusions

On the basis of the obtained results, it can be concluded that all the used raw materials (with assumed manufacture process parameters) can be used for the direct production of biocomposites on the basis of rapeseed meal made of MCC. It is useful to use an addition of chokeberry, apple, raspberry and currant pomace in the amount of 30%, which has a substantial impact on the improvement of flexural strength and the increase of water contact angle of the surface. The increase of the fruit pomace share in the samples up to 30%, regardless of the used biocomposite production temperature, causes the increase of water contact angle. The biocomposites, reinforced with an addition of chokeberry and currant pomace (addition of 20 and 30 wt%), had the best flexural strength parameters (FS) from among the tested materials, where the strength parameters ranged from 11.1 to 12.3 MPa, regardless of the used process temperature. Such results can be promising for further research associated with the limitation of the energy intensity through a reduction of the temperature in the hot-pressing process. The highest Young’s modulus of biocomposites was 1.43 GPa, which is a promising result compared to other studies. An analysis of SEM pictures showed that the irregular extended shapes (with numerous fibres) can be observed in the fracture of the tested sample, which improves physical and mechanical parameters of such materials. Generally, the increase of pomace share reduces luminosity (L*), which was observed for each analysed biocomposite. It is useful to use an addition of ChPR and BPR pomace to the biocomposite because the colour changes (Δ*E*) due to the increase of process temperature can be imperceptible with the naked eye. All the manufactured materials are characterised by the high thermal resistance between 170 and 220 °C, which is positive from the point of view of their further use, e.g., as formulated products. The use of additives of various fruit pomace (tested in this work) does not contribute to the formation of new groups of compounds. Taking into account the above, the obtained samples may have application potential, e.g., biodegradable kitchen accessories, packaging and various utility elements, and others.

## Figures and Tables

**Table 1:** Raw material composition and temperature parameters of the biocomposites production process.

Sample Acronym	Composition	Process Temperature (°C)
ChPR	(10, 20, 30 wt%) Chokeberry Pomace + (83, 73, 63 wt%) Rapeseed meal +7 wt% microcrystalline cellulose	130; 160
BPR	(10, 20, 30 wt%) Blackcurrant Pomace + (83, 73, 63 wt%) Rapeseed meal + 7 wt% microcrystalline cellulose	130; 160
APR	(10, 20, 30 wt%) Apple Pomace + (83, 73, 63 wt%) Rapeseed meal +7 wt% microcrystalline cellulose	130; 160
RPR	(10, 20, 30 wt%) Raspberry Pomace + (83, 73, 63 wt%) Rapeseed meal +7 wt% microcrystalline cellulose	130; 160
Control 1	93 wt% Rapeseed meal + 7 wt% microcrystalline cellulose	130
Control 2	93 wt% Rapeseed meal + 7 wt% microcrystalline cellulose	160
Control 3 *	93 wt% Rapeseed meal + 7 wt% microcrystalline cellulose	Δ*E* colour 130_160 °C

* Acronym for colour change Δ*E* between samples: control 1 and control 2.

**Table 2:** Average values of the particle size distribution of crushed raw materials. Average of three repetitions.

Size Range	Mass Percent (wt%)				
	Rapeseed Meal	Chokeberry Pomace	Blackcurrant Pomace	Apple Pomace	Raspberry Pomace
1.6–1.0 mm	1.94	2.45	3.08	3.57	4.60
1.0–0.8 mm	10.35	12.29	10.94	11.19	9.13
0.8–0.71 mm	5.49	6.58	7.51	7.55	6.51
0.71–0.5 mm	22.61	21.84	21.57	22.64	25.60
0.5–0.25 mm	51.73	47.88	50.11	47.76	46.92
<0.25 mm	7.01	8.19	6.61	6.90	6.49
Total mass accounted for	99.1	99.2	99.8	99.6	99.2

**Table 3:** Average values of L*, a*, b* colour changes for all biocomposites with each share of used additives. Average of five repetitions.

Samples	Process Temperature 130 °C			Process Temperature 160 °C		
	L*	a*	b*	L*	a*	b*
ChPR (10_130)	10.80 (±0.51)	−0.08 (±0.0052)	1.78 (±0.085)	9.49 (±0.81)	−0.06 (±0.0072)	1.36 (±0.095)
ChPR (20_130)	9.33 (±0.82)	−0.12 (±0.0096)	1.44 (±0.073)	8.55 (±0.53)	0.01(±0.0042)	1.13(±0.083)
ChPR (30_130)	9.04 (±0.88)	−0.04 (±0.0062)	1.38 (±0.43)	9.80 (±0.72)	−0.01(±0.0082)	1.35(±0.095)
APR (10_130)	12.09 (±0.51)	−0.08 (±0.0048)	2.06 (±0.051)	9.77(±0.63)	−0.26(±0.0078)	1.66(±0.055)
APR (20_130)	11.39 (±1.25)	0.13 (±0.0062)	2.12(±0.047)	10.24 (±0.25)	−0.13 (±0.0082)	1.67(±0.021)
APR (30_130)	11.19 (±0.42)	0.10 (±0.0092)	2.05 (±0.045)	10.07(±0.91)	−0.13 (±0.012)	1.52(±0.085)
RPR (10_130)	11.72 (±0.33)	−0.07 (±0.0031)	2.11(±0.40)	9.20 (±0.74)	−0.09(±0.0033)	1.45 (±0.11)
RPR (20_130)	11.67 (±1.12)	−0.16 (±0.0102)	2.00(±0.14)	9.40 (±0.66)	−0.08(±0.0093)	1.63(±0.091)
RPR (30_130)	10.72 (±0.91)	0.08 (±0.0022)	2.49 (±0.094)	10.28 (±0.81)	−0.18 (±0.0079)	1.68(±0.099)
BPR (10_130)	10.65 (±0.38)	0.02 (±0.001)	1.61(±0.075)	9.40 (±0.28)	−0.09 (±0.0092)	1.42(±0.088)
BPR (20_130)	9.83 (±0.43)	−0.03(±0.0012)	1.48(±0.11)	8.65(±0.64)	−0.02 (±0.002)	1.40(±0.094)
BPR (30_130)	9.63 (±0.30)	−0.04(±0.0043)	1.29(±0.025)	8.72(±0.49)	−0.06 (±0.0031)	1.28(±0.10)
Control 1	15.47(±0.13)	−0.32 (±0.0093)	2.87(±0.014)	-	-	-
Control 2	-	-	-	9.33 (±0.11)	−0.03 (±0.001)	1.31(±0.014)

## Data Availability

The data presented in this study are available on request from the corresponding author.
